# Novel Method for NTC Thermistor Production by Aerosol Co-Deposition and Combined Sintering

**DOI:** 10.3390/s19071632

**Published:** 2019-04-05

**Authors:** Michaela Schubert, Christian Münch, Sophie Schuurman, Véronique Poulain, Jaroslaw Kita, Ralf Moos

**Affiliations:** 1 Department of Functional Materials, Universität Bayreuth, 95440 Bayreuth, Germany; functional.materials@uni-bayreuth.de; 2 Vishay Electronic GmbH, Dr.-Felix-Zandman-Platz 1, 95100 Selb, Germany; CC-NLR-Division@vishay.com; 3 Vishay Resistors Belgium BVBA, Twee Huizenstraat 37, 1140 Brussel, Evere, Belgium; CC-NLR-Division@vishay.com

**Keywords:** aerosol deposition method (ADM), RTIC, NiMn_2_O_4_ ceramic, NTCR device, temperature sensor, thick film thermistor

## Abstract

A novel three-stage process to produce NTCR sensors is presented. In this process, an uncalcined powder mixture of NiO and Mn_2_O_3_ was deposited onto an alumina substrate via aerosol co-deposition (AcD). Then, an electrode structure was screen-printed onto the surface and the composite film was sintered in a multifunctional temperature treatment. Thereby, the sintering of the electrode, the formation of the NiMn_2_O_4_ spinel and the removal of film strains took place simultaneously. This enabled a significant reduction in energy demand and workload. The manufactured sensors, both as first prototypes, as well as miniaturized chip components, were characterized by a single-phase cubic NiMn_2_O_4_ spinel structure, mechanical stability and electrical properties that were similar to those of classical NiMn_2_O_4_ bulk ceramics or tempered aerosol deposited (AD) NiMn_2_O_4_ films. Particularly noteworthy was the high reproducibility and low variation of the NTCR parameters, such as the specific resistivity at 25 °C *ρ*_25_, the electrical resistance at 25 °C *R*_25_ and the thermistor constant *B*. The NTCR parameters were as aging-stable as for NiMn_2_O_4_ bulk ceramics or tempered NiMn_2_O_4_ AD-films and could even be further improved by thermal post-treatment.

## 1. Introduction

Due to their high-temperature sensitivity, reliability, and low-cost, ceramic NTC thermistors are widely used as temperature sensors in industrial, domestic and medical applications [1,2,3,4]. They are mainly based on transition metal spinel manganites (0 < *x* < 1, M: Ni, Co, Cu, etc.) [5,6], such as NiMn_2_O_4_, and are characterized by an almost exponential drop in electrical resistance with increasing temperature. The operating temperature of spinel-based NTC thermistors is in the range of −50 °C to 150 °C [7]. The high-temperature dependence of the electrical resistance *R* is based on a small polaron hopping, a conduction mechanism based on phonon-assisted hopping of charge carriers along Mn^3+^ and Mn^4+^ cations on octahedral interstices, via localized states [6,8]. The *R*-*T*-behavior can be described by the Arrhenius equation [9,10,11]:

*R*(*T*) = *R*_0_ · exp(*E*_A_/(*k*_B_ · *T*)) = *R*_0_ · exp(*B*/*T*) (1)

where *R*_0_ is the electrical resistance at infinitely high temperature and *T* is the temperature in Kelvin. The quotient of the activation energy for the hopping process *E*_A_ and the Boltzmann constant *k*_B_ is defined as the so-called *B* constant. Besides the resistivity at 25 °C *ρ*_25_, this is an important parameter to describe and compare NTCR ceramics. The commercial production of NTCR ceramic sensors is based almost exclusively on traditional, sinter-based processes [5,12]. In these processes, a pre-calcined (600 °C to 800 °C) ceramic powder is usually formed by processes such as pressing, extrusion, film casting, and eventually sintered at temperatures above 1000 °C under controlled atmospheres [2,12]. However, the thermistors produced with these processes are limited in terms of their integration capability in electronic circuit boards and miniaturization [7]. Therefore, different thin and thick film processes for the production of ceramic NTCR sensors are currently being investigated [7]. The aerosol deposition (AD) process has proven to be extremely promising. Starting from ceramic powder, dense ceramic films can be produced at room temperature and on a wide variety of substrate materials [13,14,15,16]. The films are characterized by a high density, good substrate adhesion as well as a nanocrystalline film structure and production-related film strains. Even NTC thermistors can be deposited directly from the spinel-based ceramic powder at room temperature [17,18,19,20,21]. The NTCR films are mechanically stable and exhibit NTCR parameters (*ρ*_25_, *B*), which are only slightly above those of the classic bulk ceramics, as found in References [4,5,22,23]. By a mild tempering (60 minutes at 400 °C) the NTCR parameters can even be varied slightly and reach the bulk values [19]. 

Despite the excellent thermistor properties, the process as it is described in References [18,19,24] has the disadvantage of the many process steps. As described above, a complete spinel formation is necessary, which requires a multi-stage powder preparation. It involves a combined powder mixing and milling, drying and calcining at 900 °C, followed by a second powder milling, drying and sieving. In addition, electrodes must be applied separately to the substrate, e.g., by screen-printing and subsequent electrode firing at 700 °C to 900 °C. In order to achieve thermally stable properties comparable to those of bulk ceramics, it is necessary to heat treat the produced films again to reduce film strains, increase grain size and reduce the number of grain boundaries.

A way to simplify the process is the novel method described in this paper. It combines the already known method of aerosol co-deposition (AcD), which is the deposition of a ceramic powder mixture, as described in References [25,26,27], with a multifunctional sintering step, in which the composite film is in situ calcined, tempered, and the screen-printed electrodes are sintered simultaneously. This process not only eliminates the second milling and drying step, but also combines the previous three temperature treatments (powder calcination, electrode firing, film tempering) into one thermal process step. 

In the first part of this study, the feasibility of the process is presented on the basis of first demonstrator devices, as well as accompanying XRD measurements, SEM analyses and electrical measurements. The second part of this study shows the implementation of the concept into chip-based NTC thermistor components of the size 3.2 mm × 1.6 mm (size 1206). Likewise, electrical measurements and aging tests were carried out.

## 2. Materials and Methods

**Figure 1:**
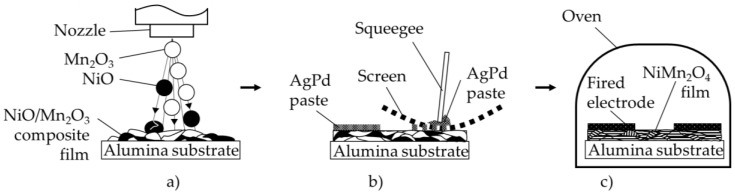
Scheme of the novel NTC thermistor production route, (**a**) aerosol co-deposition (AcD) of the powder mixture of NiO and Mn_2_O_3_; (**b**) application of an electrode structure by screen-printing; (**c**) combined electrode sintering with spinel formation and film tempering.

The initial step is the powder mixing. The raw materials, preparation equipment and preparation route are described in detail by Schubert et al. [27]. The finished powder mixture is analyzed via XRD (D8 ADVANCE, Bruker AXS, Bruker Corporation, Billerica, USA). Afterward, the first prototypes were manufactured in three steps according to the new method (see Figure 1). In the first step, as shown in Figure 1a, the aerosol co-deposition on a bare alumina substrate (Rubalit 710, CeramTec) took place. The apparatus used for the deposition process is described in detail by Hanft et al. [28]. The apparatus basically consisted of a vacuum pump, a deposition chamber and an aerosol-generating unit (here, a fluidized bed). During the process, the carrier gas oxygen with a flow rate of 6 l/min was forced through a loose filling of the powder mixture and thus an aerosol was generated. A pressure difference between the aerosol generation unit (~20 kPa) and the deposition chamber (~0.5 kPa) caused the powder aerosol to be transported into the deposition chamber. The powder aerosol passed the nozzle (0.5 × 10 mm slot nozzle) and was further accelerated to several hundred meters per second. In the deposition chamber (schematically shown in Figure 1a), suitable particles of NiO and α-Mn_2_O_3_ collided with the alumina substrate. The substrate was placed at a distance of 4–5 mm from the nozzle and was moved at a speed of 1 mm/s, perpendicular to the nozzle. On impact with the substrate, the particles deformed and/or broke up into fragments in the nanometer range. The particle fragments bonded onto each other and onto the substrate. By this deposition mechanism, they formed a firmly adherent ceramic composite film of NiO and α-Mn_2_O_3_ under the influence of other impacting particles (densification was by the hammering effect, Room Temperature Impact Consolidation (RTIC) mechanism [13]).

In the second step of the method (Figure 1b), an interdigital electrode structure (29 electrode finger pairs, finger length 4.7 mm, line/space 100 µm) was screen-printed onto the surface of the composite film using an AgPd paste (6146, DuPont). In the third and last step, the films were heated to 850 °C for 10 min. In this step, the screen-printed electrode was fired, and simultaneously, the composite film was calcined and tempered. In order to investigate the influence of the deposition process and the temperature treatment on the crystal structure, XRD analyses (PANalytical XPert Pro, PANalytical, Almelo, Netherlands) were performed in parallel to sensor manufacturing. For this purpose, the powder mixture was co-deposited under identical parameters onto a Si-wafer. The film was examined in the deposited state and in the in situ calcined state at room temperature. In addition, the spectra were evaluated with regard to the strain *ε* and the crystallite size *L* using the Williamson–Hall method:

Δ(2 *Θ*) · cos(*Θ*) = (*K* · *λ*)/(*L*) + 4 · *ε* · sin(*Θ*) (2)

where *K* is the dimensionless Scherrer constant (*K* = 0.9), *λ* is the wavelength of the X-ray source (here Cu Kα1: 1.5406 Å) and *Θ* is the Bragg angle. The reflex broadening in half of the maximum Δ(2 *Θ*) was determined via the Lorentz fit function. The prepared sensors were characterized by SEM (Leo 1450 VP, Zeiss, Oberkochen, Germany). Electrical resistance measurements were carried out (four-wire setup) in the in situ calcined state and in the aged state, in a thermostat bath (Julabo SL-12, Julabo GmbH, Seelbach, Germany) with low viscosity silicone oil (dow corning 200 fluid, 5 cst., Dow Corning Corporation, Midland, MI, USA) using a digital multimeter (Keithley 2700, Keithley Instruments Inc., Solon, OH, USA). The bath temperature was controlled by a precision Pt1000 device. The aging of the sensors took place at 125 °C for 1000 h under air. 

In order to apply the novel three-stage process (see Figure 1) for chip-based components, an AcD was performed on a laser-patterned (breaking edge) alumina substrate (Rubalit 710, CeramTec). In the second step (Figure 1b), an interdigital electrode structure (9 electrode finger pairs, finger length 2.13 mm) was applied onto the composite film by the screen-printing of an Au paste (5744R, DuPont). The distance between the electrode fingers and the finger width was 70 µm each. In the third step (Figure 1c), the multifunctional temperature treatment took place at 850 °C for 10 min. Finally, the components were separated manually. The components were electrically characterized in the in situ calcined state and in the aged state. The electrical measurement was carried out in a silicone oil bath (same set up as above) at 25 °C and 85 °C. The devices were aged for 1000 h at 125 °C after the in situ calcination and after an additional pre-treatment step at 600 °C for 60 min.

## 3. Results and Discussion

### 3.1. Characterization of the First Prototypes

**Figure 2:**
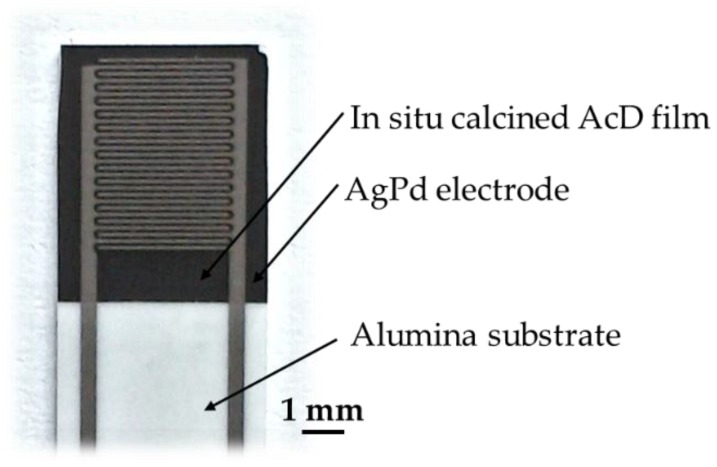
Finished NTCR sensor after completion of multifunctional temperature treatment.

Figure 2 shows an NTCR sensor after completion of the multifunctional temperature treatment. The combination of the alumina substrate, the aerosol deposited film, and the screen-printed AgPd interdigital electrode was mechanically stable. No cracks, spalling or delamination were visible. Both the electrode and the aerosol deposited NTCR film did neither peel off in the tape test nor by mechanical scratching. The electrode structure was precisely deposited and showed no short-circuits.

**Figure 3:**
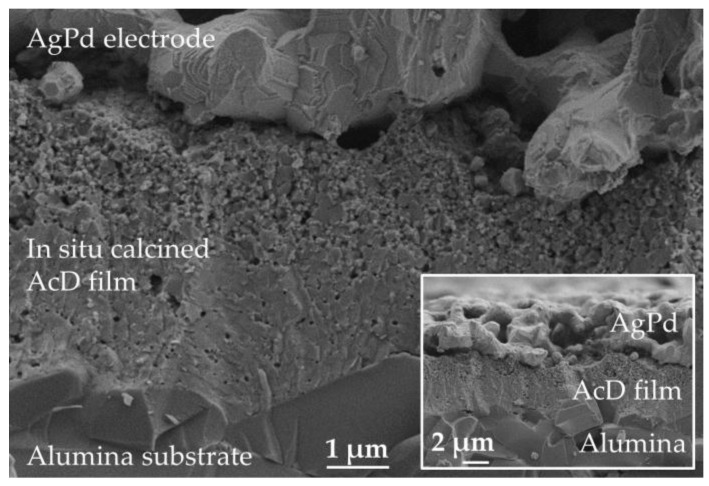
SEM analysis of the fracture surface after calcination of the sensor.

Good adhesion of the film to the substrate and to the electrode was also confirmed in the SEM analysis of the fracture surface (see Figure 3). In the lower part of the SEM image is the alumina substrate, in the middle is the AcD film, and at the upper end is the AgPd electrode. No cracks or delamination could be detected within the NTCR sensor. The adhesion of both the AcD film on the substrate and the AgPd electrode on the AcD film was very good after in situ calcination. The AgPd film has the typical structure of screen-printed films consisting of sintered metal beads. Compared to the screen-printed AgPd film, the in situ calcined AcD film was very dense. However, the calcined AcD film morphology differed noticeably from classic AD films in the as-deposited state [18,19]. Thus, in the lower half of the film, AD untypical pores in the range of 10 nm to 100 nm occurred. The upper half of the film showed a recrystallized structure of uniform, octahedral grains in the range of 100 nm, similar to grains produced via an oxalic precursor route [5].

The pores formed in the lower half of the film were due to grain growth and the release of oxygen. Oxygen was released during the intermediate reaction NiMnO_3_ + 1/2 α-Mn_2_O_3_ → NiMn_2_O_4_ + 1/4 O_2_ [23,29], and could not diffuse outwards due to the high film density. The recrystallization in the upper half of the film may have been due to sintering additives from the screen-printing paste. However, diffusion of Ag, Pd or other elements into the film could not be detected by energy dispersive X-ray (EDX).

**Figure 4:**
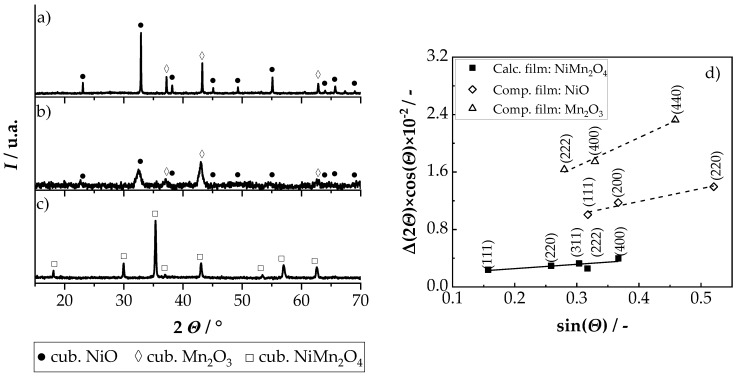
XRD spectra of (**a**) starting powders, (**b**) AcD film in the as-deposited state, (**c**) AcD film after calcination and (**d**) Williamson–Hall plots of NiO and α-Mn_2_O_3_ in composite film in the as-deposited states and NiMn_2_O_4_ after calcination of the composite film; (NiO: PDF-Nr. 01-073-1523; α-Mn_2_O_3_: PDF-Nr. 01-078-0390; NiMn_2_O_4_: PDF-Nr. 01-084-0542).

The results of the accompanying XRD analysis are shown in Figure 4. Figure 4a shows the XRD spectrum of the initial powder mixture of NiO and Mn_2_O_3_, Figure 4b shows the spectrum of the AcD film in the as-deposited state and Figure 4c shows the spectrum of the AcD film after the in situ calcination. The analysis of the three spectra (Figure 4a–c) using the Williamson–Hall method is shown in Figure 4d. It is shown that only reflections of cubic NiO (PDF-Nr. 01-073-1523) and cubic α-Mn_2_O_3_ (PDF-Nr. 01-078-0390) could be found, both in the powder mixture (Figure 4a) and in the AcD film in the as-deposited state (Figure 4b). Consequently, during the powder production and co-deposition process, there was no change in the crystal structure compared to the starting oxides. The reflexes in the AcD film (Figure 4b) were very strongly widened with low intensity. This is typical for AD films and was due to the nanocrystalline film structure and the film strains caused by the RTIC mechanism [30,31,32]. The evaluation of the strain and crystallite size using the Williamson–Hall method (see Figure 4d) gave a strain of about 0.5% and crystallite sizes of about 30 nm for NiO, and about 1% and 29 nm for Mn_2_O_3_.

This high strain and the very small crystallite size were consistent with the literature [30,33,34,35,36]. After the in situ calcination (Figure 4c), the XRD spectrum showed only the reflexes of a cubic NiMn_2_O_4_ (PDF-Nr. 01-084-0542) spinel. Thus, it could be proved that an in situ calcination of an AcD film to the desired NiMn_2_O_4_ is possible. Also, the reflexes were significantly narrower and with more intensity compared to the deposited state. As a result, internal film strains could be reduced during calcination and the crystallite size could be increased. This was also confirmed by an evaluation of the spectrum using the Williamson–Hall method (Figure 4d), which showed a crystallite size of 103 nm and a strain of only 0.1%. 

**Figure 5:**
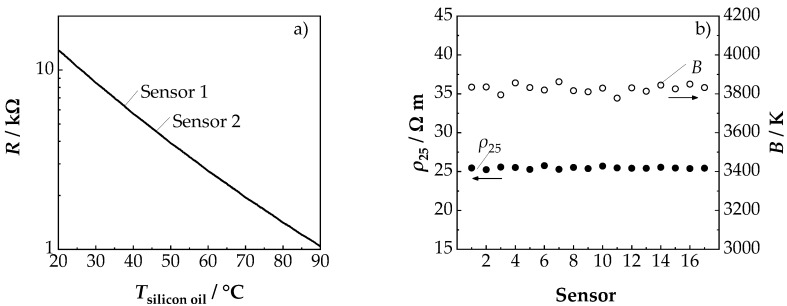
(**a**) *R*-*T* characteristics of two randomly chosen sensors (number 1 and 2 from Figure 5b) and (**b**) determined *B* and *ρ*_25_ values of all 17 produced sensors.

The single-phase cubic NiMn_2_O_4_ spinel structure and the almost completely eliminated film strains were also reflected in the NTCR characteristic achieved. Thus, the manufactured components, as demonstrated on the example of sensors 1 and 2 (Figure 5a), exhibited the typical exponential NTCR behavior. With a room temperature resistance *R*_25_ in the range of 10 kΩ, the sensors were in a range that was appropriate for typical applications. Particularly noteworthy, were the achieved NTCR parameters *ρ*_25_ and *B* in Figure 5b. With 25.5 Ω m and 3825 K, the parameters were exactly in the range of those of classic NiMn_2_O_4_ bulk ceramics (*B* = 3500 K − 3900 K, *ρ*_25_ = 20 Ω m − 30 Ω m [4,5,22,23]). This is a major advantage of the process compared to conventional AD process, in which an additional tempering step is required to reduce film strains, increase grain size and reduce the number of grain boundaries in order to achieve the bulk characteristics [17,18,19]. 

**Figure 6:**
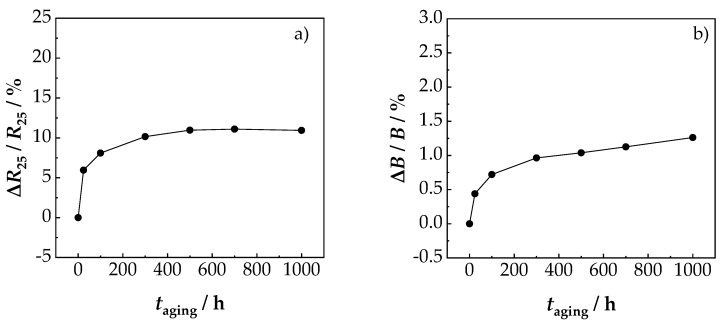
Influence of aging for 1000 h at 125 °C on (**a**) *R*_25_ value and (**b**) *B* value.

Another positive aspect was the very good reproducibility of the NTCR parameters *ρ*_25_ and *B*. As shown in Figure 5b, both the *ρ*_25_ and the *B* value of the 17 sensors scattered below ±1% around the average value. This proved the good reproducibility of the suggested novel device preparation method. The aging results of the sensors for 1000 h at 125 °C are shown in Figure 6. It could be seen that the main change of *B* and *ρ*_25_ and occurred within the first 100 h. This is typical for NTCR ceramics and has been frequently observed in the literature [5,37,38].

After aging for 1000 h at 125 °C, the *B* value changed by about 1%, and the *R*_25_ value by about 11%. Thus, the resistance increase rate due to aging was in the range of classical bulk NiMn_2_O_4_ sensors (10%–15% [5,38]) as well as in the range of tempered NiMn_2_O_4_ AD films (≤10% [24]). At higher aging temperatures, e.g., 200 °C (above the maximum operating temperature of 150 °C), a stronger aging occurs. Here too, the main change of *B* and *ρ*_25_ took place within the first 100 h. Above 400 °C, decomposition of the spinel is to be expected. For practical applications, processes such as pre-aging [2] or improved material compositions [5] are used. Based on these findings, miniaturized chip components were manufactured and tested.

### 3.2. Chip-Based NTC Thermistor Components

**Figure 7:**
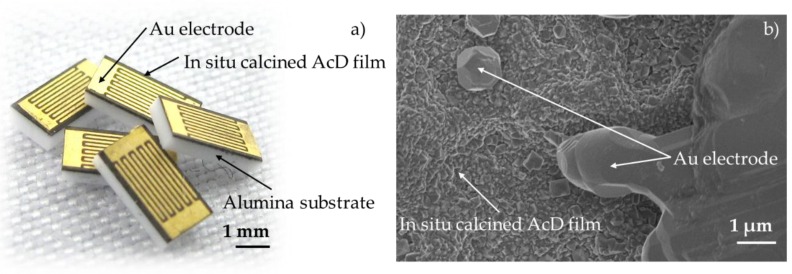
Finished chip-based NTCR sensor after completion of multifunctional temperature treatment (**a**) photograph and (**b**) SEM image of the surface.

The following Figure 7 shows a photograph and an SEM image of the surface of the completely processed chip-based NTCR sensor devices. As already stated under Section 3.1, the combination of the substrate, the AD film and the electrode was mechanically stable without cracks, spalling or delamination. The Au electrode was precisely imaged and has the intended finger width and distance of 70 µm (Figure 7a). As shown in the SEM image in Figure 7b, the in situ calcined film structure differed from that of the aerosol deposited films [28] as well. Indeed, an AD-typical crater-like surface structure [14] could be recognized. The particles were no longer strongly deformed and no nanograined structure was evident. Instead, a recrystallized microstructure with grains in the range of 100 nm to 500 nm could be seen. As already described under Section 3.1, this structure was presumably due to the sintering process and possible sintering additives from the screen-printing paste. Nevertheless, the very dense film structure has to be emphasized. At sintering temperatures of only 850 °C, this dense film structure cannot be achieved by the classic ceramic processes due to the poor sintering properties of NTCR ceramics [17,19,39,40].

**Figure 8:**
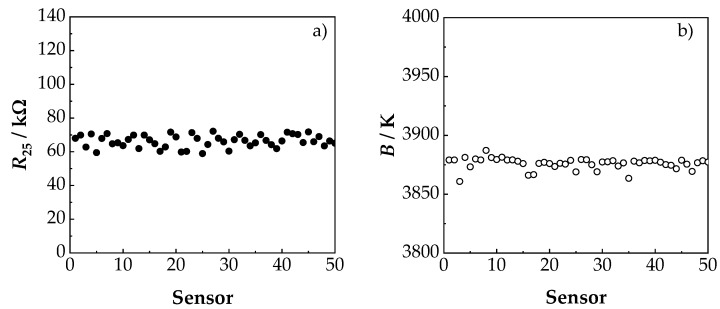
Determined (**a**) *R*_25_ value and (**b**) *B* values of 50 sensors.

So far, all sensors had been manufactured individually. Due to the miniaturized design, it is now possible to produce 50 components per deposition process. This allows statements about process noise, leading to a variation of the NTCR parameters. Figure 8 summarizes the NTCR parameters of 50 sensor components. As shown in Figure 8a, the electrical resistance of the components at 25 °C (*R*_25_) was on average 65 kΩ and had a standard deviation of 4 kΩ. The *R*_25_ value was higher than the *R*_25_ value in Section 3.1, mainly because of a different electrode structure (see Section 2). 

The dispersion in the *R*_25_ values was due to two reasons—the homogeneity of the NTCR film thickness and the precision of the screen-printed electrode. Thus, the film thickness of the aerosol co-deposited film varied slightly, both over the nozzle width and over the film length. The same applied to the electrode structure, which also fluctuated slightly due to the manufacturing process. The *B* value in Figure 8b was on average 3876 K and had a standard deviation of only 5 K. The *B* value of 3876 K was slightly higher than the *B* value of 3825 K of the prototypes (Figure 5). This difference of about 1% was mainly attributed to fluctuations in the manual powder production. 

**Figure 9:**
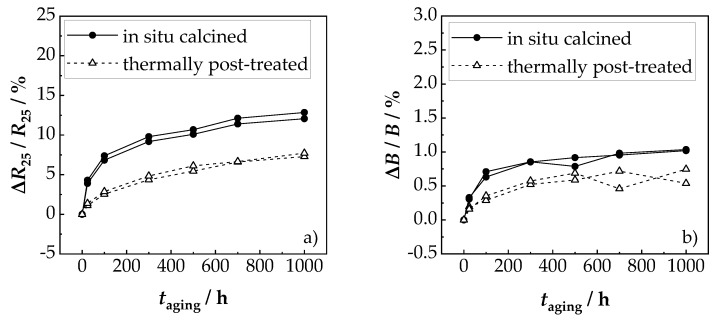
Aging-related change of (**a**) *R*_25_ value and (**b**) *B* value for chip-based components after 1000 h at 125 °C.

Considering the aging behavior of chip-based components at 125 °C, the results (Figure 9) were similar to that of the prototypes. Here too, the *R*_25_ values increased by about 12% and the *B* values by about 1%. Thus, not only the individually prepared samples but also the industrial-like manufactured samples, showed the same aging behavior of bulk-based NiMn_2_O_4_ ceramics [5,38] and tempered NiMn_2_O_4_ AD films [24]. An increase in *R*_25_ of 12% was relatively high, but not uncommon for unstabilized systems such as NiMn_2_O_4_ [38]. An improvement could be achieved when utilizing optimized compositions as described in the literature, as in References [41,42,43], or when using polyphase ceramics [5,24]. For instance, Rousset et al. [5] have already observed that a polyphase ceramic of spinel and NiO reduces aging. A similar behavior could also be found with a polyphase ceramic of NiMn_2_O_4_, NiMnO_3_ and α-Mn_2_O_3_ [24]. This polyphase ceramic (NiMn_2_O_4_ + NiMnO_3_ + α-Mn_2_O_3_) could be achieved by partial decomposition of the NiMn_2_O_4_ spinel by thermal post-treatment in the unstable range of >400 °C [44] to <730 °C [23]. 

The improved aging stability is also shown in Figure 9a,b. It could be seen that subsequently, thermally post-treated components (600 °C for 60 min) were significantly less prone to aging than purely in situ calcined components. Thus, the change in the *R*_25_ value was only 7% and the change in the *B* value was only 0.6%. Consequently, an improvement in the aging stability of aerosol co-deposited films was also possible through the formation of multi-phase ceramics.

## 4. Conclusions

In the present study, it could be shown that on the basis of a new method, consisting of only three steps—aerosol co-deposition of a powder mixture, screen-printing of an electrode structure, multifunctional temperature treatment—mechanically stable NTCR sensors could be reproducibly produced. It has been shown, by means of initial prototypes, that the NTCR parameters *ρ*_25_ and *B*, as well as their aging stability, were similar to those of classic bulk NiMn_2_O_4_ ceramics or tempered NiMn_2_O_4_ AD films. Based on this novel process route, miniaturized chip-based components were developed and manufactured. These chip-based sensors exhibited the same properties as the first prototypes. It could also be shown that the components could be manufactured with high reproducibility and that aging could be improved by thermal post-treatment to form a polyphase ceramic.
